# (CCUG)_n_ RNA toxicity in a *Drosophila* model of myotonic dystrophy type 2 (DM2) activates apoptosis

**DOI:** 10.1242/dmm.026179

**Published:** 2017-08-01

**Authors:** Vildan Betul Yenigun, Mario Sirito, Alla Amcheslavky, Tomek Czernuszewicz, Jordi Colonques-Bellmunt, Irma García-Alcover, Marzena Wojciechowska, Clare Bolduc, Zhihong Chen, Arturo López Castel, Ralf Krahe, Andreas Bergmann

**Affiliations:** 1 Department of Biochemistry & Molecular Biology, University of Texas MD Anderson Cancer Center, Houston, TX, USA; 2 Departments of Genetics, University of Texas MD Anderson Cancer Center, Houston, TX, USA; 3 Graduate Programs in Genes & Development, University of Texas Graduate School in Biomedical Sciences at Houston, Houston, TX, USA; 4 Department of Molecular, Cell and Cancer Biology, University of Massachusetts Medical School, Worcester, MA, USA; 5 Valentia BioPharma, Paterna, Spain; 6 Graduate Programs in Human & Molecular Genetics, University of Texas Graduate School in Biomedical Sciences at Houston, Houston, Texas, USA

**Keywords:** Myotonic dystrophy, DM2, RNA toxicity, *Drosophila*, Muscleblind, Apoptosis

## Abstract

The myotonic dystrophies are prototypic toxic RNA gain-of-function diseases. Myotonic dystrophy type 1 (DM1) and type 2 (DM2) are caused by different unstable, noncoding microsatellite repeat expansions – (CTG)_DM1_ in *DMPK* and (CCTG)_DM2_ in *CNBP*. Although transcription of mutant repeats into (CUG)_DM1_ or (CCUG)_DM2_ appears to be necessary and sufficient to cause disease, their pathomechanisms remain incompletely understood. To study the mechanisms of (CCUG)_DM2_ toxicity and develop a convenient model for drug screening, we generated a transgenic DM2 model in the fruit fly *Drosophila melanogaster* with (CCUG)_n_ repeats of variable length (*n*=16 and 106). Expression of noncoding (CCUG)_106_, but not (CCUG)_16_, in muscle and retinal cells led to the formation of ribonuclear foci and mis-splicing of genes implicated in DM pathology. Mis-splicing could be rescued by co-expression of human MBNL1, but not by CUGBP1 (CELF1) complementation. Flies with (CCUG)_106_ displayed strong disruption of external eye morphology and of the underlying retina. Furthermore, expression of (CCUG)_106_ in developing retinae caused a strong apoptotic response. Inhibition of apoptosis rescued the retinal disruption in (CCUG)_106_ flies. Finally, we tested two chemical compounds that have shown therapeutic potential in DM1 models. Whereas treatment of (CCUG)_106_ flies with pentamidine had no effect, treatment with a PKR inhibitor blocked both the formation of RNA foci and apoptosis in retinae of (CCUG)_106_ flies. Our data indicate that expression of expanded (CCUG)_DM2_ repeats is toxic, causing inappropriate cell death in affected fly eyes. Our *Drosophila* DM2 model might provide a convenient tool for *in vivo* drug screening.

## Abstract (precis)

**Summary:** A *Drosophila* model of myotonic dystrophy type 2 (DM2) recapitulates several features of the human disease, identifies apoptosis as a contributing factor to DM2, and is likely to provide a convenient tool for drug screening.

## INTRODUCTION

Myotonic dystrophy (DM) is the most common adult-onset neuromuscular disorder (Harper, 2001). DM is characterized by myotonia, muscle weakness and wasting, as well as multi-systemic manifestations, including insulin resistance, gonadal atrophy, cataracts and neuropsychiatric symptoms (La Spada and Taylor, 2010; Udd and Krahe, 2012; Thornton, 2014). There are two genetically distinct types, DM1 and DM2, which are caused by similar noncoding repeat expansions in different genes: a (CTG)_n_ expansion in the 3′ UTR of the DM1 protein kinase (*DMPK*) gene in DM1; and a (CCTG)_n_ expansion in the first intron of the CCHC-type zinc finger nucleic acid binding protein (*CNBP*) gene [also known as zinc finger protein 9 (*ZNF9*)] in DM2 (La Spada and Taylor, 2010; Udd and Krahe, 2012; Timchenko, 2013; Thornton, 2014). Whereas expansion size generally correlates with disease severity in DM1 and is the basis for the observed pronounced anticipation, there does not appear to be a genotype/phenotype correlation in DM2 (Udd and Krahe, 2012). DM2 expansions up to 44 kb (11,000 CCTG) have been reported (Liquori et al., 2001; Day et al., 2003; Sallinen et al., 2004); the smallest expansions associated with clinically detectable manifestations are between 55 and 100 CCTG repeats (Liquori et al., 2001; Lucchiari et al., 2008; Bachinski et al., 2009).

The prevailing paradigm is that both DM1 and DM2 are toxic RNA-mediated spliceopathies, mediated by the mutant expansions of normally polymorphic (CTG)_n_ or (CCTG)_n_ repeats: transcription into (CUG)_DM1_ or (CCUG)_DM2_ RNA is necessary and sufficient to cause disease (Osborne and Thornton, 2006; Klein et al., 2011; Sicot and Gomes-Pereira, 2013). Mutant RNAs accumulate in ribonuclear foci and interfere with RNA splicing, transcription and/or translation of downstream effector genes, resulting in the characteristic pleiotropic phenotype (Schoser and Timchenko, 2010; Jones et al., 2011; Sicot et al., 2011; Udd and Krahe, 2012; Timchenko, 2013).

Mechanistically, (CUG)_DM1_ or (CCUG)_DM2_ RNA foci sequester Muscleblind-like (MBNL) proteins, which are zinc-finger RNA-binding proteins involved in alternative RNA splicing (Miller et al., 2000; Mankodi et al., 2001, 2003; Kanadia et al., 2003; Pascual et al., 2006; Lee and Cooper, 2009; Schoser and Timchenko, 2010; Jones et al., 2011; Meola et al., 2013). MBNL proteins are highly conserved from flies to humans. The fruit fly *Drosophila melanogaster* has a single MBNL gene, *muscleblind* (*mbl*), which is involved in muscle development and photoreceptor neuron differentiation in the eye (Begemann et al., 1997; Artero et al., 1998; Pascual et al., 2006). Loss of *mbl* causes muscle defects and blindness, hence the name of the gene (Begemann et al., 1997; Artero et al., 1998). Similarly, in DM1 and DM2 patients [humans have three MBNL homologous genes: *MBNL1-3* (Fardaei et al., 2002)], the sequestration of MBNL proteins in RNA foci reduces the amount of functional MBNL proteins available for proper splicing, resulting in a shift from the normal adult splice pattern to an inappropriate embryonic/fetal pattern of target transcripts (Miller et al., 2000; Mankodi et al., 2001; Jiang et al., 2004; Kanadia et al., 2006; Holt et al., 2009).

More than 20 transcripts have been shown to be mis-spliced in DM (Jiang et al., 2004; Gatchel and Zoghbi, 2005; Botta et al., 2007; Du et al., 2010). For example, aberrant splicing of the muscle-specific chloride channel *CLCN1* and the insulin receptor (*INSR*) accounts for myotonia in DM (Savkur et al., 2001, 2004; Mankodi et al., 2002; Wheeler et al., 2007; Tonevitsky and Trushkin, 2009; Tang et al., 2012; Santoro et al., 2013). Other mis-spliced genes in DM include the muscle contractile proteins cardiac troponin (*TNNT2*) and skeletal muscle troponin (*TNNT3*) (Philips et al., 1998; Yuan et al., 2007; Vihola et al., 2010).

In addition to the MBNL family of proteins, at least two other RNA-binding proteins have been implicated in DM1. Expanded CUG repeats increase the activities of CUG-binding protein (CUGBP1; also known as CELF1) and dsRNA-dependent protein kinase (PKR; also known as EIF2AK2) (Tian et al., 2000; Timchenko et al., 2001a,b; Mankodi et al., 2003; Ward et al., 2010; Jones et al., 2011). Whether these factors are involved in DM2 is unclear.

There is currently no cure for DM. Most efforts to identify therapeutic modes of intervention are focused on the reversal of RNA toxicity. To develop a convenient model for drug screening, we generated a DM2 model in the fruit fly *Drosophila melanogaster*. We obtained transgenes that express noncoding transcripts of variable size, with the largest at 106 CCUG repeats (*DM2*-*106*). Transgenic *DM2*-*106* flies recapitulate many features observed in the human disease condition. They form RNA foci in muscles and retinal cells and affect RNA splicing of splicing reporter genes. Although we did not observe muscle atrophy in *DM2*-*106* flies, they displayed strong disruption in the external morphology of the eye and underlying retina. Expression of MBNL1, but not CUGBP1, was able to rescue the eye phenotype of *DM2*-*106* flies. Furthermore, *DM2*-*106* flies exhibited a strong apoptotic response in developing retinae, and inhibition of apoptosis rescued the retinal disruption. Finally, we tested two chemical compounds with therapeutic potential in DM1. Whereas treatment of *DM2*-*106* flies with pentamidine had no effect, treatment with a PKR inhibitor blocked both the formation of RNA foci and apoptosis in retinae of *DM2*-*106* flies. These data suggest that the *Drosophila* DM2 model described here may provide a suitable tool for drug screening.

## RESULTS

### Transcripts with expanded (CCUG)_n_ repeats form RNA foci

**Fig. 1.**
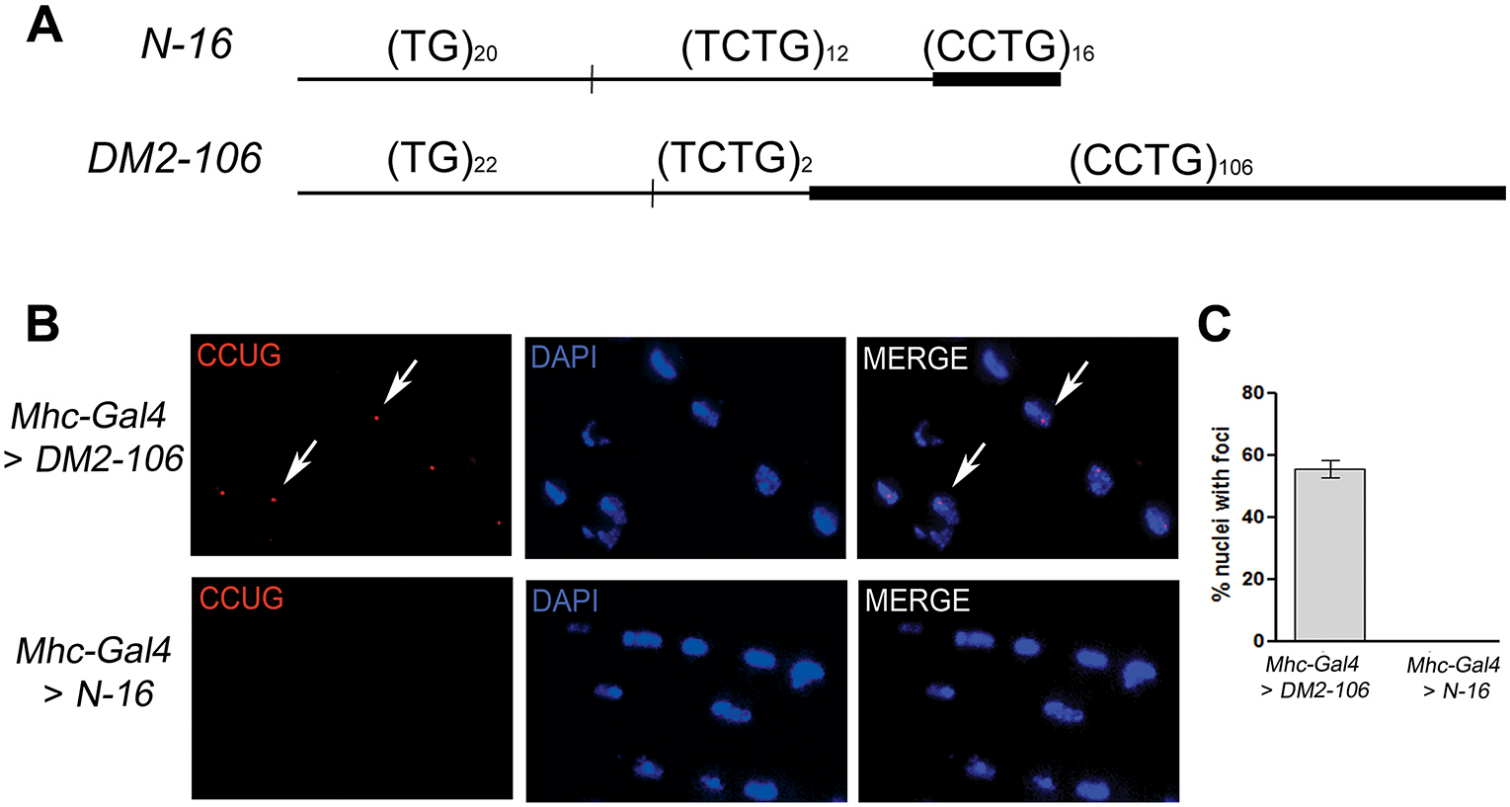
**A *Drosophila* DM2 model forms nuclear CCUG foci.** (A) Schematic (not to scale) of the noncoding CCTG repeat constructs used in this study. The control contains (CCTG)_16_ repeats (*N-16*), which is non-toxic in humans. The mutant construct contains (CCTG)_106_ repeats (*DM2-106*). Both constructs are preceded by polymorphic (TG)_n_(TCTG)_n_ repeats, as indicated, that are also part of the complex human repeat motif. These constructs are under control of the UAS promotor. (B) *In situ* hybridization using a locked nucleic acid (LNA) probe was performed on 15 μm cryosections of thoracic muscles of flies expressing *DM2-106* and control repeats using the myosin *Mhc-Gal4* driver. *DM2-106* expression is associated with the presence of ribonuclear foci (red) in DAPI-stained nuclei (blue), whereas no foci are detected in controls using the same *Gal4* driver. Two representative foci are indicated (arrows). (C) Quantification of nuclei with ribonuclear foci in control and *DM2-106* muscle cells using *Mhc-Gal4*. Error bars indicate s.d.

The smallest reported DM2 expansions associated with clinically detectable manifestations are between 55 and 100 CCTG repeats (Liquori et al., 2001; Lucchiari et al., 2008; Bachinski et al., 2009). To generate a DM2 model in *Drosophila*, we prepared two transgenes: a control transgene expressing a noncoding transcript with 16 CCUG repeats in the normal range (referred to as *N-16*), and an experimental transgene expressing a noncoding RNA with 106 CCUG repeats (*DM2-106*) (Fig. 1A). Because the (CCTG)_DM2_ expansion is part of a complex polymorphic motif (Bachinski et al., 2003, 2009) of the form (TG)_n_(TCTG)_n_(CCTG)_>26_ and the (TG)_n_(TCTG)_n_ polymorphic repeats have been shown to affect DNA structure (Edwards et al., 2009), we included a (TG)_n_(TCTG)_n_ tract in our (CCTG)_DM2_ constructs. Both control and DM2 transgenes contained the polymorphic (TG)_n_(TCTG)_n_ repeats upstream of the (CCTG)_n_ tract: the *N-16* allele had a (TG)_20_(TCTG)_12_(CCTG)_16_ motif, while the *DM2-106* allele had a (TG)_22_(TCTG)_2_(CCTG)_106_ motif (Fig. 1A). These transgenes are under the control of a UAS promoter (Brand and Perrimon, 1993) and expression can be induced using convenient Gal4 drivers, such as muscle-specific *Mhc*-*Gal4* and eye-specific *GMR*-*Gal4*.

Because myotonia and muscle wasting are associated with human DM2, we first expressed the control and disease transgenes using *Mhc*-*Gal4* and analyzed the morphology of the indirect flight muscle (IFM). As nuclear retention of RNA-protein aggregates (foci) is a hallmark of DM2 (Mankodi et al., 2003; Jones et al., 2011; Udd and Krahe, 2012; Meola et al., 2013), we first determined that *DM2*-*106* flies mirror this disease-linked trait and performed FISH analysis to detect foci in the nucleus of IFM cells of *DM2*-*106* flies. No foci were detected in control IFM, whereas more than 50% of the cells analyzed had nuclear foci in *DM2*-*106* flies (Fig. 1B,C), demonstrating that 106 CCUG repeats are sufficient to cause biochemical changes. The average fraction of nuclei with ribonuclear foci in *DM2*-*106* muscle cells is similar to that observed in a DM1 fly model expressing 480 CTG repeats (García-Alcover et al., 2014).

### Expression of *DM2*-*106* in *Drosophila* muscles causes mis-splicing

**Fig. 2.**
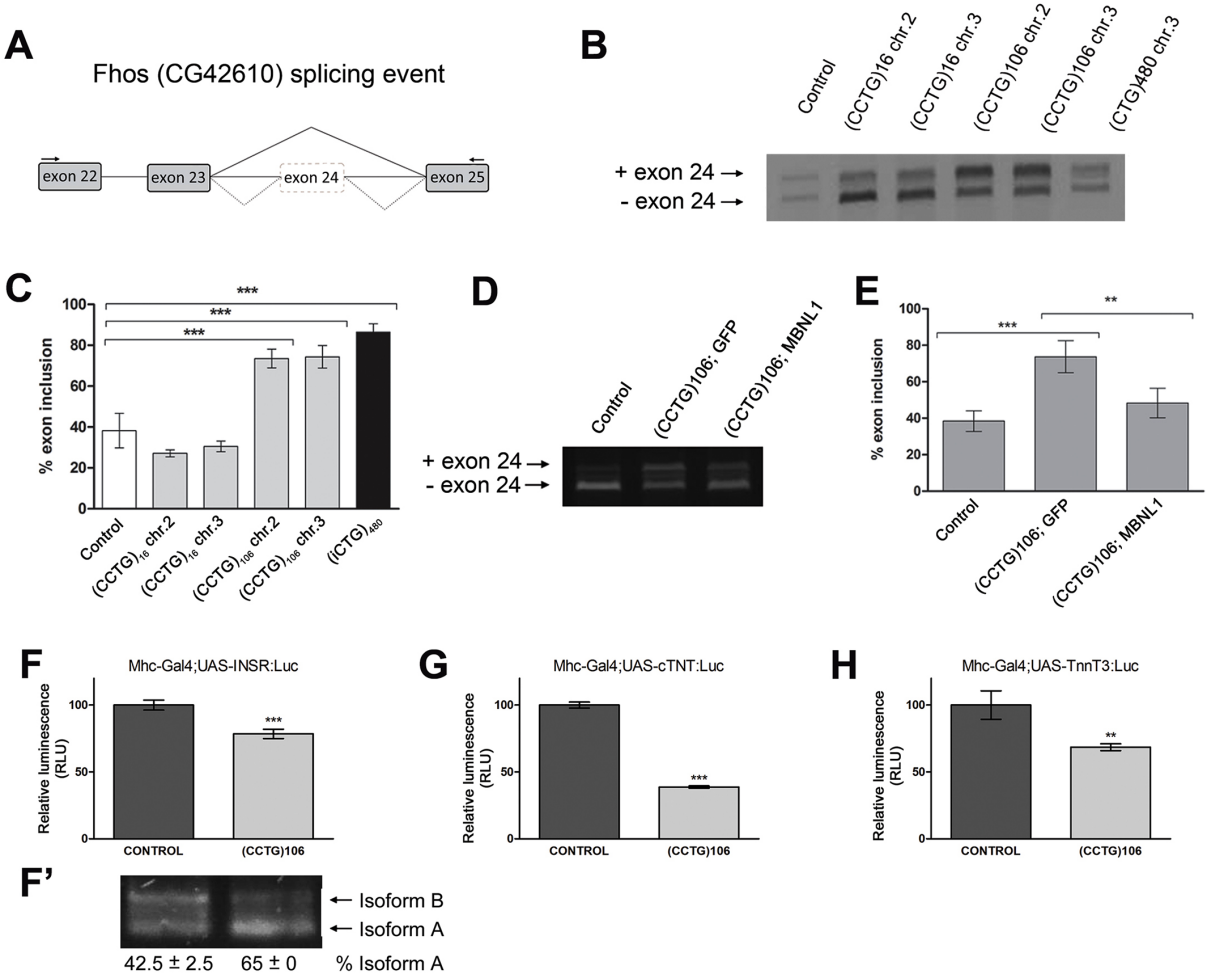
***DM2-106* expression in muscle causes mis-splicing of MBNL1-dependent transcripts.** (A) Outline of the intron/exon structure of *Fhos* (*CG42610*) showing the exons implicated in the splicing event studied. Wild-type flies mainly skipped exon 24 (solid line), whereas *DM2-106* expression in IFM led to aberrant inclusion of exon 24 (dotted lines). Arrows indicate primers used for semi-quantitative PCR analysis. (B,C) Agarose gel and quantification of *Fhos* RT-PCR products from IFM expressing control (*N-16*) and *DM2-106* transgenes located on chromosomes 2 and 3. These transgenes were driven by *Mhc-Gal4*. Flies that only contain the *Mhc-Gal4* driver without a UAS transgene show an average frequency of exon 24 inclusion of ∼30%. Compared with this control, expression of normal repeat length (CCUG)_16_ does not significantly alter *Fhos* splicing, whereas in the (CCUG)_106_ repeat-expressing cells exon 24 is retained at ∼70%, levels similar to those of DM1 flies expressing an interrupted 480 CUG repeat sequence (iCUG)_480_. (D,E) Agarose gel and quantification of *Fhos* RT-PCR products from flies expressing the indicated transgenes with the *Mhc-Gal4* driver. Simultaneous expression of human *MBNL1* and *DM2-106* induces exon 24 exclusion, restoring wild-type levels (*Mhc-Gal4* only). Error bars represent s.d. and each experiment was repeated at least twice in adults of 0-5 days of age. (F-H) Luminescence levels of *Mhc-Gal4*>*UAS*-*minigene,DM2-106* normalized to the levels of *Mhc-Gal4*>*UAS*-*minigene,UAS*-*GFP*. Relative luminescence decreased from 100% in control flies to 78% for the human *INSR* reporter minigene (F), 38% for *TNNT2* (*cTNT*) (G) and 68% for mouse *Tnnt3* (H). RLU, relative light units. **P<0.005, ***P<0.001 (Student's *t*-test). (F′) RT-PCR analysis of the *INSR* spliceosensor in *N-16* and *DM2*-106 background. The percentage is the average of two experiments.

In order to evaluate *DM2*-*106* flies as a suitable DM2 model, we examined mis-splicing events in transgenic flies expressing the 106 CCUG repeats in IFM. We studied alternative splicing of the endogenous *Fhos* gene (Fig. 2A), which showed aberrant splicing regulation in DM1 flies expressing a (CTG)_480_ tract (Garcia-Lopez et al., 2008) (see also Fig. 2B). For this analysis, we used two different transgenes for control and *DM2*-*106* constructs, located on chromosomes 2 and 3. Expression of both *DM2*-*106* transgenes increased the frequency at which exon 24 was aberrantly included (Fig. 2B): quantification revealed an increase from ∼30% in *N-16* control flies to >70% in *DM2*-*106* flies (Fig. 2C), similar to DM1.

The MBNL proteins are sequestered in (CCUG)_DM2_ foci and have been implicated as important mediators of DM2-associated spliceopathy. To validate an involvement of MBNL factors in DM2 flies, we co-expressed the human *MBNL1* gene and *DM2*-*106* in *Drosophila* IFM. As shown in Fig. 2D, *MBNL1* expression rescued exon 24 inclusion levels in IFM in the presence of (CCUG)_106_, unlike GFP protein, which was used as a negative control in this co-expression experiment. The frequency of disease-linked exon 24 inclusion was reduced from 73% (*DM2*-*106*+*GFP*) to 48% (*DM2*-*106*+*MBNL1*), close to control levels in the non-disease situation (38%) (Fig. 2E).

The suitability of our *Drosophila* DM2 system as a disease model was further demonstrated by the observation that different spliceosensor luciferase reporters, which express specific mammalian reporter mini-genes for identified mis-splicing events in DM1 and DM2 (human *INSR* exon 11 and mouse *Tnnt3* fetal exon) (Savkur et al., 2004; Vihola et al., 2010; García-Alcover et al., 2014), were also responsive to the presence of expanded CCUG repeats (Fig. 2F,H). In addition, we tested a *TNNT2* exon 5 spliceosensor reporter that shows mis-splicing in DM1 (Philips et al., 1998). All three spliceosensor reporters revealed alternative splicing aberrations, resulting in reduced luciferase luminescence, when (CCUG)_106_ repeats were expressed in the IFM (Fig. 2F-H). To verify that the significant changes in luciferase luminescence were due to mis-splicing, we examined the splicing pattern of the *INSR* spliceosensor reporter directly by RT-PCR in our DM2 fly model. In the *Drosophila* DM1 model, two splice isoforms of the *INSR* spliceosensor were detectable due to the inclusion (isoform B) or exclusion (isoform A) of an alternative exon between exons 11 and 12 (García-Alcover et al., 2014). Isoform A is preferentially observed in the disease state (García-Alcover et al., 2014). In the *N-16* controls expressing (CCUG)_16_, isoform A was present at 42.5% (Fig. 2F′), consistent with the previous report by García-Alcover et al. (2014). By contrast, in the presence of expanded (CCUG)_106_ repeats (*DM2-106*), the relative amount of isoform A increased to 65% (Fig. 2F′), which correlated with the decreased luciferase luminescence observed in Fig. 2F. These results demonstrated that *DM2*-*106* transgenic flies display a spliceopathy phenotype similar to that seen in human DM2 patients and thus validate it as a suitable DM2 model.

### Expression of (CCUG)_106_ in *Drosophila* IFM does not cause muscle atrophy

**Fig. 3.**
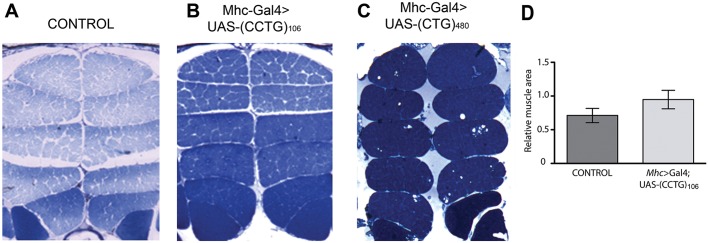
***DM2*-*106* expression does not cause morphological defects in *Drosophila* musculature.** (A-C) IFM transverse sections from control flies (*Mhc-Gal4*/+) or flies expressing either (CCUG)_106_ (*DM2-106*) or (CUG)_480_ (DM1). (CUG)_480_ expression leads to vacuolization and muscle disorganization (Garcia-Lopez et al., 2008), whereas (CCUG)_106_ expression was not disruptive to muscle fiber morphology. (D) Relative muscle areas of at least six independent thoraces of each genotype were calculated after binarization using ImageJ and statistically analyzed using a two-tailed, non-paired *t*-test (*P*=0.118). Error bars indicate s.d.

To study the extent of (CCUG)_DM2_ toxicity in our DM2 model, we analyzed IFM samples expressing control or expanded (CCUG)_n_ (*N-16* or *DM2*-*106*) for morphological defects similar to those described in patients (Vihola et al., 2003; Bassez et al., 2008). However, in contrast to the phenotypic alterations of the IFM in DM1 models (Fig. 3C), no significant differences were observed between the IFMs of control and expanded (CCUG)_n_-expressing flies (Fig. 3A,B, quantified in Fig. 3D). *DM2*-*106* flies appeared to be able to fly normally and even aged flies (40 days) did not display any obvious flight defects. These results demonstrated that although (CCUG)_106_ repeats are sufficient to cause biochemical abnormalities, they are not sufficient to cause morphological and behavioral phenotypes in the IFM.

### Expanded (CCUG)_106_ repeats cause severe disorganization of eye morphology, which is modified by loss or gain of MBNL proteins

**Fig. 4.**
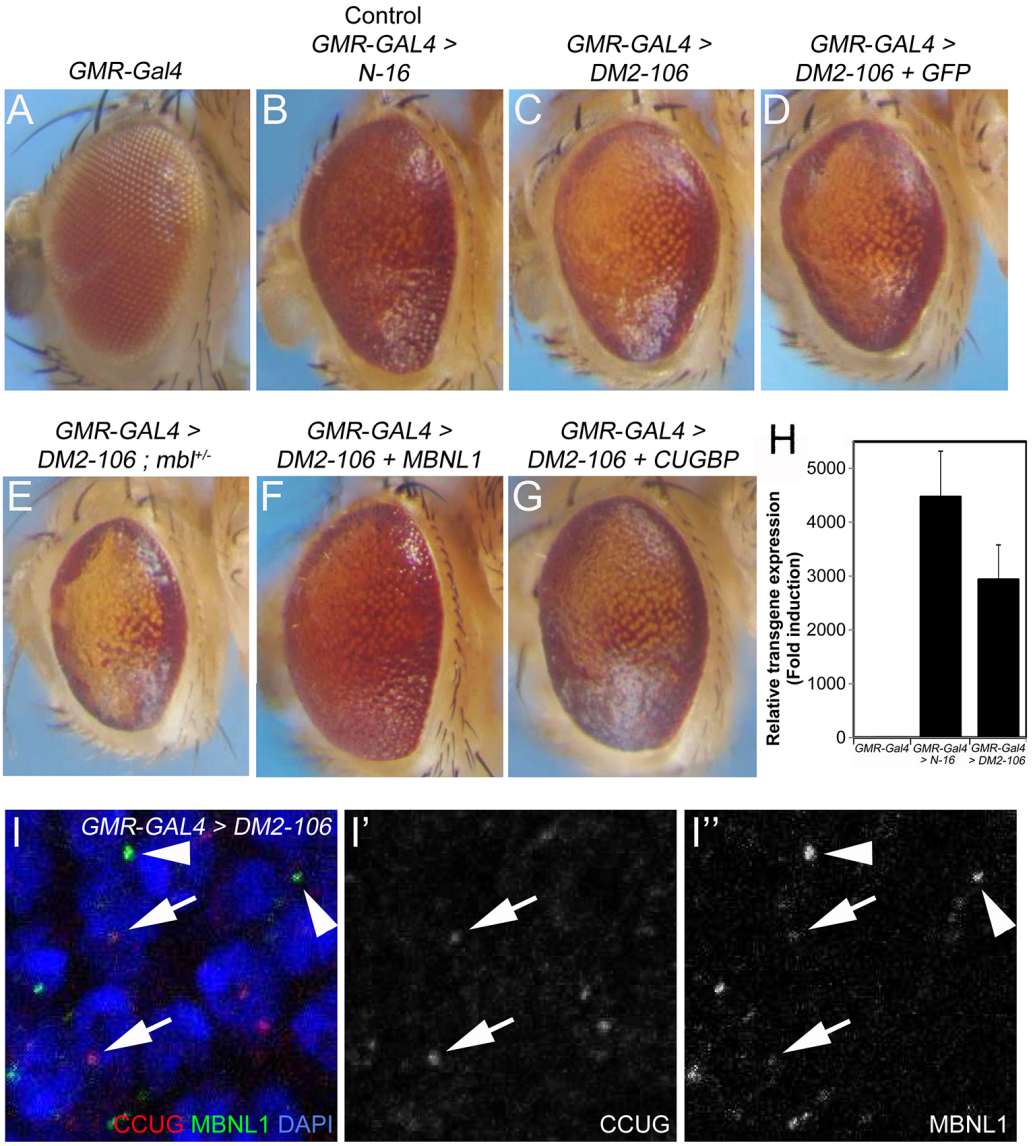
**Expression of expanded (CCUG)_n_ causes severe disruption of eye morphology, which can be modified by loss or gain of MBNL.** (A) The eye of a *GMR-Gal4*-only fly. (B) Expression of the control *N-16* transgene under the *GMR-Gal4* driver shows a very mild eye roughening, which is caused by *GMR-Gal4*. (C) Disruption of the external eye morphology of a fly that expresses *DM2-106* using *GMR*-*Gal4*. (D) Expression of a UAS control transgene, *UAS-GFP*, does not rescue the eye phenotype of *GMR-Gal4>DM2-106* flies. (E) Heterozygosity for a null allele of *muscleblind* (*mbl^KG08885^*), resulting in functional hemizygosity, severely enhances the *DM2-106* eye phenotype owing to the 50% reduction in MBL protein levels. (F) Expression of human *MBNL1* rescues the eye phenotype of *DM2-106* flies under *GMR-Gal4* control. (G) Co-expression of a *CUGBP1* transgene using *GMR-Gal4* does not rescue or provides only very little rescue of the external eye morphology of *DM2-106* flies. (H) Expression levels of control (*N-16*) and experimental (*DM2-106*) transgenes under *GMR*-*Gal4* control in eye imaginal discs. Statistical analysis was by two-tailed, non-paired *t*-test (*P*=0.222). (I-I″) MBNL1 protein (green) accumulates in CCUG foci (red) in *GMR*-*Gal4*>*DM2-106* eye imaginal discs (arrows). Interestingly, MBNL1 also aggregates in foci independently of CCUG repeats (arrowheads). Nuclei are labeled with DAPI (blue).

Because (CCUG)_106_-expressing flies did not show significant phenotypic alterations in the IFM, we turned our focus to a different phenotype commonly observed in DM2 pathogenesis, namely ocular manifestations. For that purpose, we expressed control (*N-16*) and *DM2-106* transgenes using *GMR-Gal4* in the posterior half of developing eye imaginal discs, which form the retina during late larval and pupal stages. Adult flies expressing the (CCUG)_106_ transgenes developed eyes of severely disorganized morphology (Fig. 4C). By contrast, control *GMR-Gal4>N-16* flies displayed only mildly rough eyes, as compared with *GMR-Gal4*-only eyes (Fig. 4A,B). Both transgenes are expressed at similar levels (Fig. 4H). Thus, in contrast to the IFM, expression of expanded (CCUG)_106_ repeats caused severe phenotypic abnormalities in the fly eye. A similar observation has recently been reported for a different DM2 fly model (Yu et al., 2015).

As described above, MBNL proteins are thought to be involved in DM2 pathogenesis. To confirm this in our DM2 fly model, we examined whether alterations to the gene dose of *Drosophila mbl* would modify the *DM2-106* phenotype. In a heterozygous *mbl*^+/−^ background, the eye phenotype of adult *DM2-106* flies is severely enhanced (Fig. 4E). The eyes are rougher and more disorganized, and often also reduced in size. Phenotypic rescue is observed when the human *MBNL1* protein is overexpressed in *DM2-106* flies, which suppressed the *DM2-106* eye phenotype (Fig. 4F). The eyes appear almost normal and ommatidial integrity is visible. Expression of a control UAS transgene, *UAS-GFP*, does not affect the *DM2-106* eye phenotype (Fig. 4D), suggesting that the rescue by *MBNL1* expression is not due to the additional UAS transgene. Immunolocalization reveals that MBNL1 protein is localized in CCUG foci (Fig. 4I-I″, arrows), but we also observe aggregates of MBNL1 protein outside of CCUG foci (Fig. 4I,I″, arrowheads).

Another protein implicated in the pathology of DM1 is CUGBP1 (Timchenko et al., 2001a,b; de Haro et al., 2006; Jones et al., 2011; Timchenko, 2013). However, in contrast to MBNL1, expression of human *CUGBP1* has little or no effect on the morphology of *DM2-106* eyes (compare Fig. 4G with 4C).

### *DM2-106* causes severe disruption of retinal organization

**Fig. 5.**
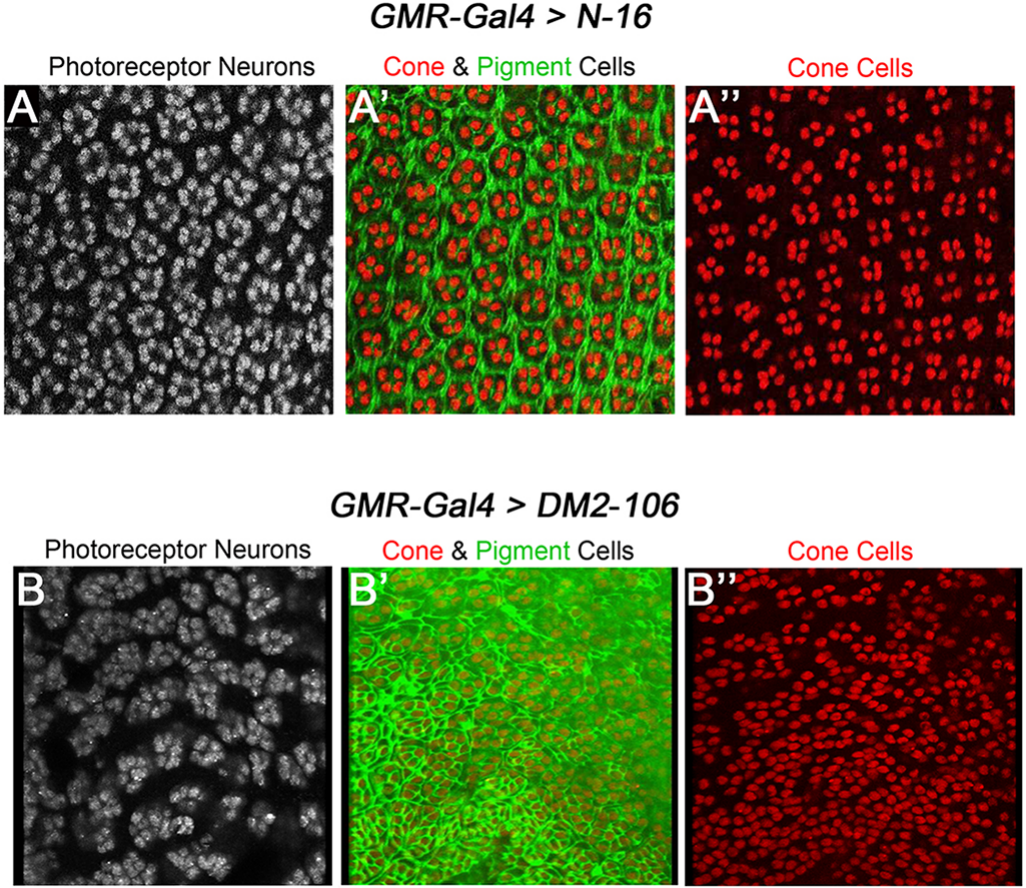
***DM2-106* causes severe disruption of retinal morphology.** Fully differentiated retinae of pupal eye imaginal discs at 42 h APF labeled with antibodies against ELAV (a marker for photoreceptor neurons; A,B), Cut (a marker of cone cells; A′,A″,B′,B″) and Dlg (to visualize cell outline and thus reveal pigment cells; A′,B′). (A′,B′) Double labeling for Cut and Dlg. (A-A″) *GMR*>*N-16* control retina showing the regular pattern of photoreceptors (A), cone (A′,A″) and pigment cells (A′). (B-B″) Retina expressing *DM2-106* under *GMR*-*Gal4* control shows irregularities of photoreceptor neurons (B), cone and pigment cells (B′,B″).

To further characterize the eye phenotype of *GMR*>*DM2*-*106* flies, we examined the underlying retinal morphology. The developing retina is fully differentiated at 42 h after puparium formation (APF). In wild-type retinae at that stage, photoreceptor neurons, cone cells and pigment cells are highly organized in a stereotypical pattern to form the individual ommatidia. Each ommatidium contains four concentrically aligned cone cells surrounded by pigment cells (Fig. 5A″). In *GMR-Gal4*>*N-16* control flies, this pattern is not significantly disturbed (Fig. 5A-A″). By contrast, in *GMR*>*DM2-106* retinae the precise cellular arrangement is severely disrupted, with photoreceptor neurons, cone cells and pigment cells irregularly positioned and numbered (Fig. 5B-B″). Ommatidia were fused and ommatidial identity was not observed. Thus, expression of (CCUG)_106_ RNA caused severe disruption of retinal morphology.

### Apoptosis induced by *DM2-106* causes retinal disruption and disorganization

**Fig. 6.**
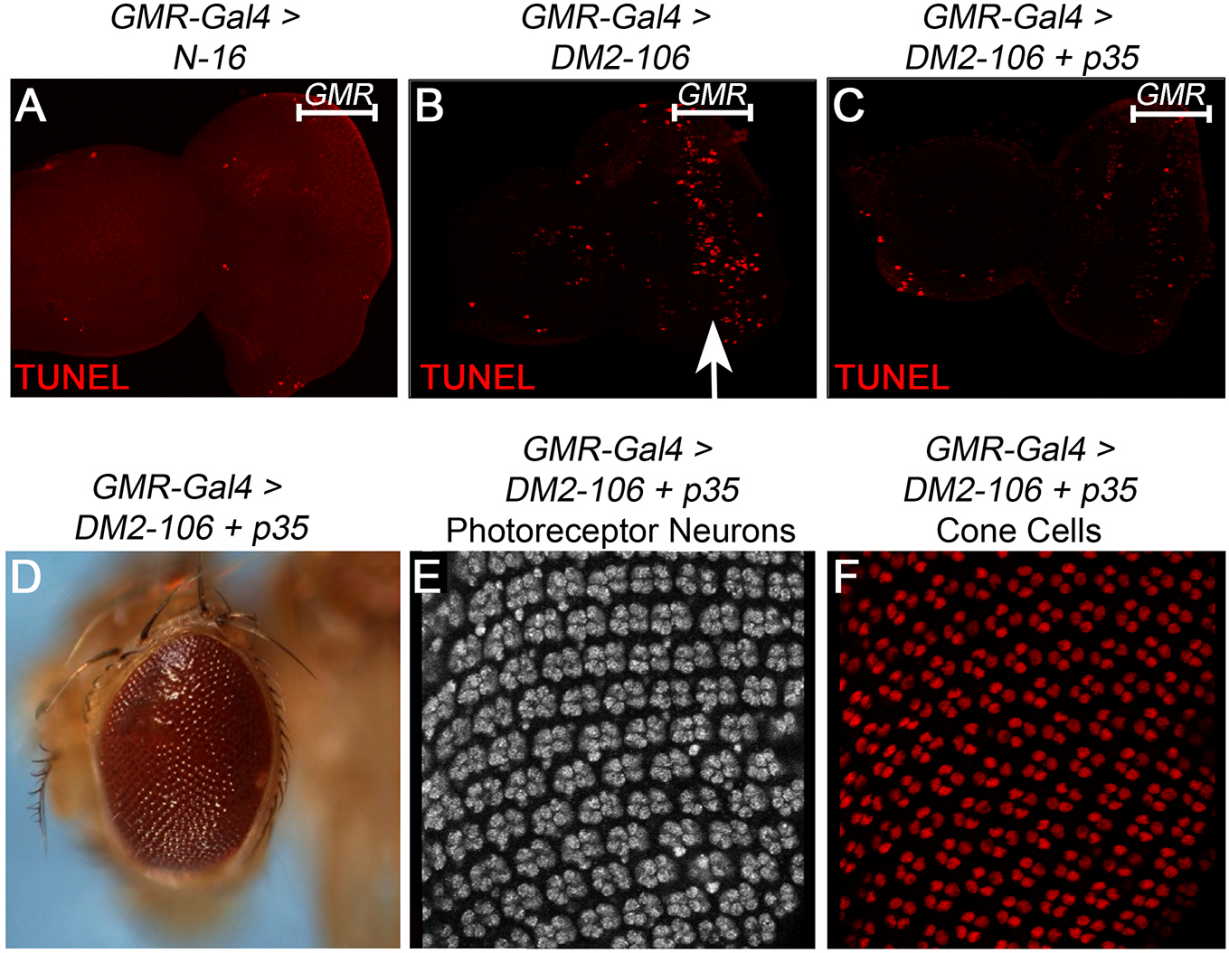
**Induction of apoptosis results in disruption of the photoreceptor neuron pattern in *DM2-106* retinae.** (A-C) TUNEL labeling as a marker for apoptosis of *N-16* (A), *DM2-106* (B) and *DM2-106*+*p35* (C) eye imaginal discs from third instar larvae under *GMR-Gal4* control. The extent of the *GMR* expression domain in the larval eye disc is indicated. The arrow (B) highlights the induced apoptosis in the posterior part of the larval eye disc where *GMR*-*Gal4* is expressed. (D) Rescue of the external eye morphology of adult *DM2-106* flies expressing the caspase inhibitor *p35* under *GMR-Gal4* control. (E,F) *GMR*>*DM2-106*+*p35* pupal retinae at 42 h APF labeled for the photoreceptor marker ELAV (E) and the cone cell marker Cut (F). Inhibition of apoptosis by co-expression of the caspase inhibitor P35 normalizes the photoreceptor and cone cell pattern in *GMR*>*DM2-106* retinae (compare with Fig. 5B).

Recently, apoptosis has been implicated in muscle degeneration in a *Drosophila* DM1 model (Bargiela et al., 2015). Therefore, we examined whether apoptosis contributes to the retinal phenotype in GMR>*DM2-106* transgenic flies. In *GMR-Gal4*>*N-16* control flies, no or very little apoptosis occurs in eye imaginal discs, the larval precursors of the adult eyes (Fig. 6A). However, in *GMR*>*DM2-106* eye imaginal discs, apoptosis is strongly induced in the *GMR* domain in the posterior half of the larval disc (Fig. 6B, arrow), suggesting that (CCUG)_106_ RNA triggers apoptosis. P35 is a potent inhibitor of apoptosis, and specifically inhibits effector caspases in flies (Hay et al., 1994; Hawkins et al., 2000; Meier et al., 2000). Co-expression of *p35* together with *DM2-106* under *GMR*-*Gal4* control suppressed the apoptotic phenotype in larval eye imaginal discs (Fig. 6C). Because P35 potently suppressed apoptosis in *DM2-106* flies, we were able to dissect the relative contribution of apoptosis to the *DM2-106* retinal and eye morphology phenotypes. Co-expression of *p35* in the *DM2-106* model normalized the external eye morphology of adult flies (Fig. 6D, compare with Fig. 4C). Furthermore, co-expression of *p35* suppressed the misalignment of photoreceptor neurons and cone cells, restoring ommatidial integrity (Fig. 6E,F, compare with Fig. 5B). These data illustrate that expression of the *DM2-106* transcript caused apoptosis that resulted in retinal disruption and disorganization.

### Feasibility of the *DM2-106* model for chemical screening

**Fig. 7.**
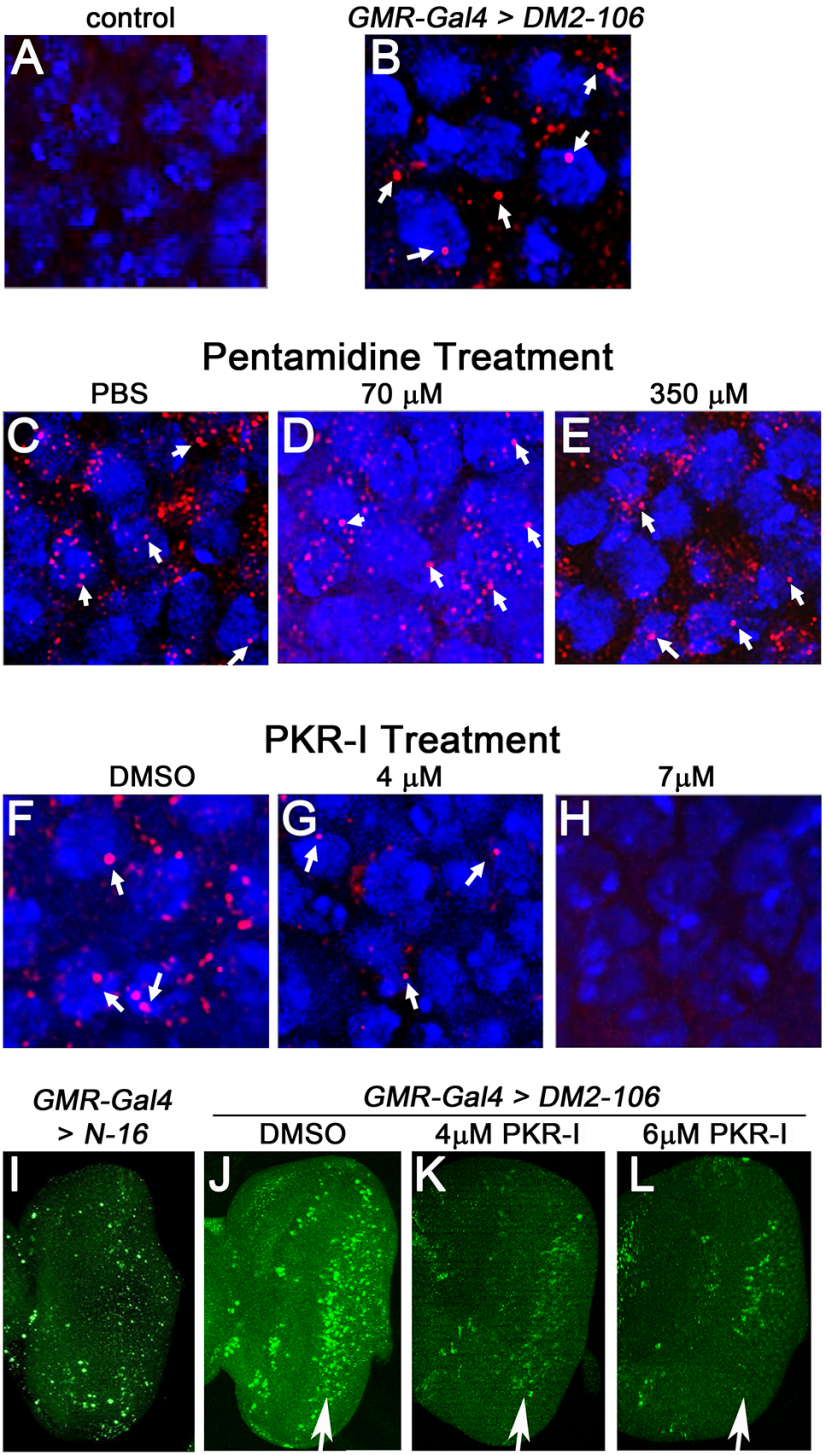
**Treatment with PKR inhibitor, but not pentamidine, blocks foci formation and apoptosis in *DM2*-*106* retinae.** (A-H) Shown are 42 h APF retinae from control (A), untreated *GMR*>*DM2-106* (B) and *GMR*>*DM2-106* treated with various buffers and drugs (C-H) as indicated. These retinae were labeled for CCUG foci (red) and nuclei (blue). Arrows indicate example foci, both nuclear and cytoplasmic. Whereas pentamidine treatment did not block foci formation up to a concentration of 350 μM, treatment with PKR inhibitor (oxindole/imidazole derivative C16, PKR-I) suppressed foci formation in a concentration-dependent manner. (I-L) Eye imaginal discs from control (I), untreated *GMR*>*DM2-106* (J) and *GMR*>*DM2-106* treated with the indicated concentrations of PKR-I (K,L). Eye discs were obtained from third instar larvae and were labeled by TUNEL as an apoptotic marker. Arrows indicate apoptosis in the GMR-expression area.

The data presented here suggest that expression of *DM2-106* in the *Drosophila* retina mimics pathological manifestations seen in the human condition, including the formation of toxic CCUG foci, as well as retinal disorganization and degeneration. Therefore, *DM2-106* might provide a suitable and convenient model for drug screening and identification of lead compounds (García-Alcover et al., 2013). To assess the feasibility of our *DM2-106* model for drug screening, we tested two compounds that have previously been shown to have therapeutic potential in DM1 models. Pentamidine is a dsRNA-intercalating drug that was found to disrupt the MBNL1-CUG repeat complex in DM1 (Warf et al., 2009). It was recently reported that pentamidine treatment can also rescue cardiac dysfunction in a *Drosophila* DM1 model (Chakraborty et al., 2015). The second drug, the oxindole/imidazole derivative C16, is an inhibitor of the dsRNA-dependent protein kinase PKR (PKR-I), which is activated by expanded CUG repeats in DM1 (Tian et al., 2000, 2005; Huichalaf et al., 2010; Wojciechowska et al., 2014). As an assay for drug treatment, we examined the ability of the selected inhibitors to block the formation of toxic CCUG foci in the *DM2*-*106*-expressing retina (Fig. 7B). Interestingly, these foci were not only nuclear, but could also be observed in the cytoplasm (Fig. 7B, arrows). Pentamidine treatment up to 350 μM, a concentration that has been shown to be effective in DM1 (Warf et al., 2009), has no visible effect on RNA foci formation in the *DM2-106* model (Fig. 7C-E). By contrast, treatment with PKR-I showed a pronounced decrease in the abundance of CCUG RNA foci in a concentration-dependent manner: 4 μM PKR-I caused a significant reduction of RNA foci, and 7 μM completely disrupted foci formation (Fig. 7F-H), resembling wild-type retinae (Fig. 7A). Consistently, loss of CCUG foci by PKR-I treatment correlated with reduction and loss of apoptosis (Fig. 7I-L). These examples illustrate that the *DM2*-*106* retina might provide a convenient model for drug screening in flies.

**Fig. 8.**
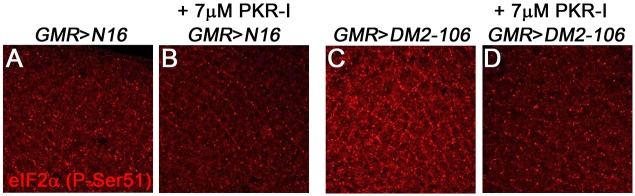
**Expression of pathogenic (CCUG)_106_ increases PKR activity in retinae of *GMR*>*DM2-106* flies.** The posterior portions of eye imaginal discs from third instar larvae of the indicated genotype labeled with phospho-specific eIF2α (P-Ser51) antibody to detect PKR activity. *GMR-Gal4>UAS-N-16* (A,B) or *GMR-Gal4*>*UAS-DM2-106* (C,D) discs were treated (B,D) or not (A,C) with 7 μM PKR-I.

The suppression of foci formation and apoptosis by PKR-I suggests that PKR activity is increased in the retinae of *DM2-106* expressing pathogenic CCUG repeats. Activation of PKR by expanded CUG repeats in DM1 has been reported (Tian et al., 2000; Huichalaf et al., 2010; Wojciechowska et al., 2014). It is currently unknown whether PKR is also activated in DM2. To examine this possibility, we tested a known PKR phosphorylation target, eukaryotic translation initiation factor 2 alpha (eIF2α) (Proud, 2005). In humans, PKR phosphorylates and inactivates eIF2α on Ser51 (Proud, 2005). This phosphorylation site is conserved in *Drosophila* eIF2α, and phospho-specific eIF2α (P-Ser51) antibodies cross-react with phosphorylated *Drosophila* eIF2α (Williams et al., 2001; Farny et al., 2009). These antibodies detect a strong increase in eIF2α phosphorylation on Ser51 in *DM2-106* retinae as compared with the *N-16* control (Fig. 8A-C). Importantly, this strong increase in Ser51 phosphorylation in *DM2-106* retinae was significantly reduced upon dietary administration of 7 μM PKR-I (Fig. 8D). These data suggest that PKR activity is strongly induced in the presence of 106 CCUG repeats in the retina.

## DISCUSSION

The goal of this work was to develop a fly model that can be used for drug screening to identify therapeutic compounds for potential treatment of DM2 patients. The rationale was that many cell biological processes, including apoptosis, alternative RNA splicing and the genes/proteins involved, are highly conserved between flies and humans. Furthermore, the genetic tools available in *Drosophila* allow for rapid characterization of the underlying phenotypes. Finally, experimentation with flies is relatively inexpensive and the short generation time enables rapid genetic and chemical screening.

Here, we describe a DM2 fly model that expresses 106 CCUG repeats in a non-coding transcript (*DM2*-*106*). Several features of *DM2*-*106* flies indicate that expression of expanded (CCUG)_106_ RNA elicits molecular and cellular phenotypes similar to those associated with DM2 pathology in human patients. *DM2*-*106* transcripts aggregate in RNA foci that are predominantly nuclear, but can also be observed in the cytoplasm, at least in retinal cells. Cytoplasmic (CCUG)_DM2_ foci have also recently been described in human HeLa cells (Jones et al., 2015). These RNA foci sequester MBNL proteins, which causes mis-splicing in muscles similar to that seen in human DM2 patients. Although we did not observe muscle atrophy in *DM2*-*106* flies, the retinae and eyes of these flies were severely disrupted. Functional complementation by overexpression of human MBNL1 protein in *GMR*>*DM2*-*106* rescued the retinal degeneration. Furthermore, inhibition of apoptosis restored the retinal pattern and eye morphology, suggesting that expression of (CCUG)_106_ in *DM2*-*106* flies induced apoptosis as the underlying cause of the retinal degeneration. The involvement of apoptosis in retinal degeneration is consistent with the recent finding that apoptosis also contributes to muscle degeneration in a *Drosophila* DM1 model (Bargiela et al., 2015). In a pilot drug screening experiment in *DM2*-*106* eyes, we found that an inhibitor of PKR activity efficiently blocked formation of RNA foci and apoptosis, whereas pentamidine failed to inhibit foci formation. Finally, we show that pathogenic (CCUG)_n_ DM2 repeat expansions activate the dsRNA-dependent protein kinase PKR, similar to previous reports in DM1 (Tian et al., 2000; Huichalaf et al., 2010; Wojciechowska et al., 2014). Taken together, these data suggest that our *DM2*-*106* fly model provides a convenient tool for drug screening.

While this work was under way, another group published a different DM2 model in *Drosophila* (Yu et al., 2015). These authors were able to express more than 700 CCUG repeats in flies. Consistent with our observations, expression of (CCUG)_700_ repeats caused retinal and eye disruption. Interestingly, muscle atrophy was also not reported (Yu et al., 2015), suggesting that in *Drosophila* the eye is perhaps more sensitive to RNA perturbations than skeletal muscle. In this context, it is worth noting that muscle weakness and atrophy are generally weaker in DM2 patients than in DM1 (Udd and Krahe, 2012). Alternatively, it is possible that there is an expansion threshold that underlies tissue-specific manifestations of the overall DM2 phenotype and that the expression of (CCUG)_106_ repeats is insufficient in itself to induce muscle phenotypes due to mis-splicing, but could involve the recently identified RAN (repeat-associated non-ATG) translation as another pathomechanism (Zu et al., 2011).

Despite the fact that DM1 and DM2 share many pathological manifestations, they are not identical diseases (Udd and Krahe, 2012). For example, they affect different types of muscle and the neurological symptoms in DM2 are generally less severe (Thornton, 2014; Ulane et al., 2014). We also observed differences between the DM1 and DM2 models in *Drosophila*. Whereas expression of CUGBP1 enhanced the DM1 phenotype (de Haro et al., 2006), it had no obvious effect on the eye phenotype of *GMR*>*DM2*-*106* flies. Furthermore, pentamidine treatment, which was shown to be effective in DM1 (Chakraborty et al., 2015), had no effect on foci formation in *DM2*-*106*. Therefore, comparative analysis of DM1 and DM2 fly models might reveal additional differences that underlie the two diseases and thereby provide important insights into the etiology of the human phenotypes.

Our pilot drug screen revealed that the *DM2-106 Drosophila* model is well suited for drug screening. Treatment of *DM2*-*106* flies with increasing concentrations of a PKR inhibitor disrupted CCUG RNA foci formation and apoptosis in eye imaginal discs, the larval precursor tissue of adult retinae and eyes. PKR encodes a dsRNA-dependent protein kinase, which was found to be activated in DM1. Our data suggest that PKR activity is also induced by expanded (CCUG)_n_ DM2 repeats. Unfortunately, although PKR-I feeding of larvae disrupted CCUG foci formation in *GMR*>*DM2*-*106* eye imaginal discs, the resulting eye phenotype of adult flies was not rescued (Fig. S1). A possible explanation for this observation is that flies stop feeding after the larval stage, so that during pupal stages the eye phenotype can still develop. Nevertheless, we are confident that modeling of DM2 in *Drosophila* will further contribute to our understanding of the pathology of DM2 and provide an excellent platform for genetic and chemical (drug) screening.

## MATERIALS AND METHODS

### Generation of control and expanded (CCUG)_n_ repeat expression clones

The (CCTG)_DM2_ expansion is part of a complex polymorphic motif (Bachinski et al., 2003, 2009) of the form (TG)_12-26_(TCTG)_7-12_(CCTG)_3-9_(G/TCTG)_0-4_(CCTG)_4-15_. DM2 expansions can be as large as 40 kb with the CCTG motif uninterrupted (Liquori et al., 2001; Bachinski et al., 2003; Sallinen et al., 2004). Reported normal alleles have repeat tract lengths of up to 26 CCTG motifs with one or more interruptions (Bachinski et al., 2009). The smallest reported DM2 expansions associated with clinically detectable manifestations are between 55 and 100 CCTG repeats (Liquori et al., 2001; Lucchiari et al., 2008; Bachinski et al., 2009). Because this complex polymorphic repeat motif has been shown to have an effect on DNA structure (Edwards et al., 2009), we included the (TG)_n_(TCTG)_n_ tracts in the (CCTG)_DM2_ constructs. We took advantage of the repeat-primed PCR (RP-PCR) assay developed in our laboratory (R.K.) for the diagnostic detection of the DM2 expansions (Sallinen et al., 2004; Bachinski et al., 2009). Using this approach, we amplified repeats from a clinically affected, genetically confirmed DM2 patient to produce (TG)_n_(TCTG)_n_(CCTG)_n_ repeats with 16 to 189 pure (CCTG)_n_ motifs. Cloned repeats were verified by sequencing to ensure purity of the expanded (CCTG)_n_ repeat tract. In order to express the (CCTG)_n_ repeats in *Drosophila*, mutant fragments containing 106 repeats with the upstream region were recovered from the TOPO vector and cloned into pUAST (Brand and Perrimon, 1993). The same cloning procedure was used with genomic DNA from a normal individual to generate the control vector containing a normal (CCTG)_16_ allele. The presence and the length of the (CCTG)_n_ repeats in the pUAST vector were confirmed by sequencing in both directions: *DM2-106*, (TG)_22_(TCTG)_2_(CCTG)_106_; and *N-16*, (TG)_20_(TCTG)_12_(CCTG)_16_.

### Generation of the MBNL1 expression clone

The human *MBNL1* clone was obtained from OriGene (TrueClone accession number NM_021038.3). The plasmid was digested with *Not*I, to separate the insert from the vector, and with *Spe*I, to decrease the vector size and distinguish it from the insert. The *Not*I insert with the entire coding sequence for *MBNL1* was then cloned into the *Not*I site of the pUAST vector. Proper orientation was confirmed by restriction enzyme digestion and sequence analysis from both ends.

### Generation of the CUGBP1 expression clone

The human *CUGBP1* clone was obtained from OriGene (TrueClone accession number NM_006560.2). This variant is the predominant transcript and encodes isoform 1. To generate the expression clone, we used the same procedure as for the MBNL1 expression clone, except for the *Spe*I digestion, since insert and vector were readily distinguishable by size in gel electrophoresis.

### Fly husbandry

Flies were raised on normal corn agar and crosses were incubated at 25°C. The following mutants and transgenic stocks were used: *UAS-[CCTG]_16_* (control); *UAS-[CCTG]_106_* (*DM2-106*); *UAS-MBNL1*; *UAS-CUGBP1*; *UAS-p35*; *Mhc-Gal4*; *GMR-Gal4*; *mbl^KG08885^*. Generation and management of DM1 spliceosensor flies was as described (García-Alcover et al., 2014). To simplify crosses, *DM2-106* transgenes on chromosome 2 or 3 were recombined with *GMR-Gal4* on the same chromosome to yield *GMR*>*DM2*-*106* on chromosome 2 or 3. Fly eyes were photographed using a Zeiss Axio Imager Z1 compound microscope.

### Drug treatment

Fly food was supplemented with drugs at the final concentrations indicated in Figs 7, 8 and Fig. S1. Pentamidine was obtained from Sigma-Aldrich (439843) and PKR-I from Calbiochem (527451). Because PKR-I needs to be dissolved in 100% DMSO, a DMSO-only control was also performed. The same volume of DMSO-containing solutions was mixed into the food.

### Reverse transcription PCR (RT-PCR) analysis

For *Fhos* and *INSR* splicing assays, total RNA was extracted from ∼50 adult flies with Tri Reagent (Sigma) following the manufacturer's instructions. Contaminating DNA was degraded by RNase-free DNase I (Thermo Scientific). Reverse transcription was performed with SuperScript II reverse transcriptase (Invitrogen) following the manufacturer's guidelines. GoTaq polymerase (Promega) was used for PCR amplification with primers (5′-3′) Fhos-F (GTCATGGAGTCGAGCAGTGA) and Fhos-R (TGTGATGCGGGTATCTACGA), or with primers INSR-F (ACGTTTGAGGATTACCTGCACAA) and INSR-R (GAGATGGCCTGGAACGACAG), in each case for 29 cycles, with an annealing temperature of 60°C (García-Alcover et al., 2014). Band intensity was quantified using ImageJ (NIH).

For quantification of *N-16* and *DM2-106* transcript levels (Fig. 4H), total RNA was extracted from *Drosophila* eye imaginal discs using TRIzol reagent (Invitrogen). cDNA conversion was performed using the SuperScript II RNase H-Reverse Transcriptase Kit (Invitrogen). Quantitative PCR (qPCR) was performed using cDNA template and SYBR Green Power Mix (Applied Biosystems). Three sets of primers flanking the CCUG repeats were designed in the pUAST vector used to clone the transgenes: primer set #1, Fwd GTGGTGGAATGCCTTTAAT and Rev GGAGGAGTAGAATGTTGAGA; primer set #2, Fwd AAAGAAGAGAAAGGTAGAAGAC and Rev AGCAAAGCAAGCAAGAG; primer set #3, Fwd CTAGTGATGATGATGAGGCTACT and Rev TAGCAATTCTGAAGGAAAGTC. Transcript levels of *Ribosomal protein 49* (*Rp49*; also known as *RpL32*) were used for normalization across samples, using primers Fwd ACCAGCTTCAAGATGACCATCC and Rev CTTGTTCGATCCGTAACCGATG.

### Fluorescence *in situ* hybridization (FISH)

FISH analysis was performed as described (Salisbury et al., 2009), except that *Drosophila* tissue was used. Imaginal discs were imaged by confocal microscopy. RNA-FISH analysis of drug-treated retinae was performed with a (CUGG)_10_ probe.

For muscle preparation, thoraces of 0- to 5-day-old *MHC-Gal4*>*UAS-(CCTG)_106_* or *MHC-Gal4*>*UAS-(CCTG)_16_* females were dissected, embedded in OCT (Fisher HealthCare), frozen in liquid nitrogen and stored at −80°C until processed. At least five 40× magnification images of different focal planes along the *z*-axis were taken using a Leica DM2500 microscope for DAPI (UV channel) and Cy3 (green channel). The *z*-planes were stacked using Photoshop (Adobe) and the number of nuclei with foci counted with ImageJ software. At least 50 cells from each individual were counted and at least three individuals were analyzed for each compound. The percentage of cells with foci was compared between *MHC-Gal4*>*UAS-(CCTG)_106_* and *MHC-Gal4*>*UAS-(CCTG)_16_*.

### Luciferase readout

Three 0- to 5-day-old adult flies were placed in each well of a flat-bottom 96-well plate (Daslab, Barcelona, Spain) and homogenized in 150 μl 1× reporter lysis buffer (Promega). Then, 50 μl of the homogenate was transferred to a new white 96-well plate (Sterilin). Lysate luminescence was measured with an Envision plate reader (PerkinElmer) after dispensing 15 μl Luciferase Assay Reagent (Promega) with the Envision injector. At least 60 wells were analyzed for each genotype studied.

### Muscle histology

*Drosophila* thoraces (7-12 days old) were embedded in Epon for semi-thin transverse sectioning as previously described (Tomlinson and Ready, 1987). Relative muscle areas of at least six different thoraces were calculated as described (Garcia-Lopez et al., 2011).

### Immunohistochemistry

At least 20 imaginal discs per experiment were dissected from late third instar larvae and pupal retinae from 42-h-old pupae. They were fixed and stained using standard protocols (Fogarty and Bergmann, 2014). TUNEL was performed using a TUNEL assay kit (Roche Life Sciences) according to the manufacturer's instructions. Antibodies to the following primary antigens were used: ELAV [rat; 1:50; Developmental Studies Hybridoma Bank (DSHB)]; Cut (mouse; 1:50; DSHB); Dlg (rabbit; 1:100; from Kwang-Wook Choi, Korea Advanced Institute of Science and Technology, Daejeon, South Korea); MBNL1 (rabbit; 1:2000; from Charles Thornton, University of Rochester Medical Center, Rochester, NY, USA); cleaved Caspase 3 (rabbit; 1:200; Cell Signaling Technology, 9661); and eIF2α (P-Ser51) (rabbit; 1:100; Cell Signaling Technology, 3597). Secondary antibodies were donkey Fab fragments from Jackson ImmunoResearch (715-166-151, 711-096-152, 712-606-153, 711-166-152; all at 1:600). Nuclei were visualized by Hoechst and DAPI staining. Fluorescent images were taken with an Olympus Optical FV500 confocal microscope.
